# Anncolvar: Approximation of Complex Collective Variables by Artificial Neural Networks for Analysis and Biasing of Molecular Simulations

**DOI:** 10.3389/fmolb.2019.00025

**Published:** 2019-04-18

**Authors:** Dalibor Trapl, Izabela Horvacanin, Vaclav Mareska, Furkan Ozcelik, Gozde Unal, Vojtech Spiwok

**Affiliations:** ^1^Department of Biochemistry and Microbiology, University of Chemistry and Technology in Prague, Prague, Czechia; ^2^Faculty of Science, University of Zagreb, Zagreb, Croatia; ^3^Computer Engineering Department, Istanbul Technical University, Istanbul, Turkey

**Keywords:** metadynamics, neural networks, molecular dynamics simulation, collective variables, free energy simulations

## Abstract

The state of a molecular system can be described in terms of collective variables. These low-dimensional descriptors of molecular structure can be used to monitor the state of the simulation, to calculate free energy profiles or to accelerate rare events by a bias potential or a bias force. Frequent calculation of some complex collective variables may slow down the simulation or analysis of trajectories. Moreover, many collective variables cannot be explicitly calculated for newly sampled structures. In order to address this problem, we developed a new package called *anncolvar*. This package makes it possible to build and train an artificial neural network model that approximates a collective variable. It can be used to generate an input for the open-source enhanced sampling simulation PLUMED package, so the collective variable can be monitored and biased by methods available in this program. The computational efficiency and the accuracy of *anncolvar* are demonstrated on selected molecular systems (cyclooctane derivative, Trp-cage miniprotein) and selected collective variables (Isomap, molecular surface area).

## Introduction

Molecular dynamics simulation makes it possible to simulate any molecular process at the atomic level. In principle, structural and thermodynamical properties of a protein can be predicted by simulation of its folding and unfolding. Similarly, structure and stability of a protein-ligand complex can be predicted by simulation of binding and unbinding. Unfortunately, many molecular processes either cannot be simulated or their simulation is far from routine due to enormous computational costs of the molecular dynamics simulation method.

Several enhanced sampling methods have been developed in order to address this problem (Spiwok et al., 2015a). Some of these methods, such as umbrella sampling (Torrie and Valleau, 1977) or metadynamics (Laio and Parrinello, 2002), use a bias potential or a bias force to destabilize frequently sampled states and to enhance sampling of poorly sampled states. Tempering methods enhance sampling by means of elevated temperature (Abrams and Bussi, 2014). There are methods combining tempering and biasing as well as methods based on completely different principles.

Biased simulations usually require one or more preselected degrees of freedom on which the bias force or potential is applied. These degrees of freedom are referred to as collective variables (CVs). There are two technical prerequisites for CVs to be applicable in biased simulations. Firstly, a CV must be a function of atomic coordinates of the molecular system, i.e., it must be possible to calculate the value of a CV at every step of the simulation solely from atomic coordinates. Secondly, it must be possible to convert the force acting on the CV into forces acting on individual atoms, i.e., it must be possible to calculate the first derivative of the CV with respect to atomic Cartesian coordinates. Beside these technical prerequisites, in order to efficiently enhance sampling it is necessary to cover all slow motions in the molecular systems by few CVs.

There are many promising CVs that do not fulfill these requirements and therefore cannot be directly used in biased simulations. These include, for example, the results of non-linear dimensionality reduction methods (Das et al., 2006). There are examples of other CVs that fulfill these requirements; however, their calculation is computationally expensive. In order to make biased simulation with these CVs possible, we and others introduced approximations tailored for biased simulations (Branduardi et al., 2007; Spiwok and Králová, 2011; Spiwok et al., 2015b; Pazúriková et al., 2017).

Recent development of neural network algorithms allows the usage of artificial neural networks for the purpose of CV approximation. The advantage of neural networks is the fact that many of them are trained by the backpropagation algorithm (Goodfellow et al., 2016), which requires easy calculation of the derivatives of the output with respect to the input. This is exactly what is needed to convert forces acting on a CV into forces acting on atoms. Application of neural network models may also benefit from the current development of neural networks, which has lead to a number of new toolkits and programs.

**Figure 1:**
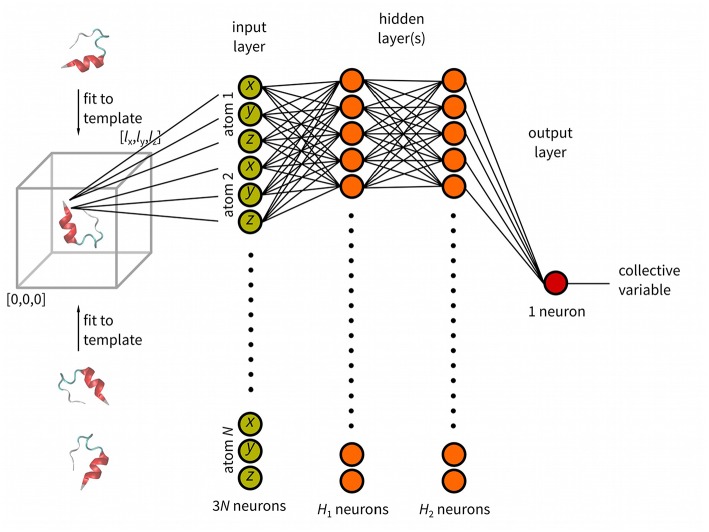
Schematic representation of *anncolvar* function. Three input files are needed for training: (i). reference structure (in PDB) of the molecule located in the center of box with one corner with coordinates [0, 0, 0] and size of [*l*_*x*_, *l*_*y*_, *l*_*z*_], (ii) training trajectory (without molecules broken by periodic boundary condition) and, (iii) file containing precomputed values of the CV for each snapshot of the training trajectory. The program generates the input file for PLUMED. In PLUMED the molecule is fit to the template (reference structure) and the CV is calculated by neural network.

Multiple recent studies have tested machine learning approaches to design collective variables for biased simulation to study thermodynamics and kinetics of molecular transitions (Galvelis and Sugita, 2017; Chen and Ferguson, 2018; Guo et al., 2018; Mardt et al., 2018; Pérez et al., 2018; Seo et al., 2018; Sultan and Pande, 2018; Wehmeyer and Noé, 2018). In this work we describe a new tool *anncolvar* for approximation of an arbitrary CV. Its function is outlined in Figure 1. This tool requires a set of structures, either a simulation trajectory or any other set of structures. For the sake of simplicity we will call this set a training trajectory. It must be accompanied with precomputed values of CVs. These data are used to train a simple neural network to approximate the value of CVs for other out-of-sample structures. It generates an input to a popular enhanced sampling program PLUMED (Bonomi et al., 2009; Tribello et al., 2014). The CV approximated by *anncolvar* can be calculated *a posteriori* for any 3D structure or trajectory. Furthermore, it can be used in metadynamics or other enhanced sampling methods available in PLUMED. This approach was tested on conformational changes of a cyclooctane derivative and Trp-cage mini-protein folding. Isomap (Tenenbaum et al., 2000) low-dimensional embeddings used as CVs in the metadynamics simulation of the former system represent CVs that cannot be calculated explicitly from Cartesian coordinates. Solvent-accessible surface area (SASA) used as a CV in simulations of the later system represents a CV that can be calculated explicitly from Cartesian coordinates, but such calculation is slow.

The program can be accessed for free at https://github.com/spiwokv/anncolvar or via PyPI.

## Methods

### Use of Anncolvar

The program *anncolvar* is written in *Python* and uses packages *mdtraj* (McGibbon et al., 2015), *numpy* (Oliphant, 2006) and *keras* (Cholet, 2018)^1^. The machine learning package *keras* runs on top of one of three machine learning backends, namely *TensorFlow, Theano* or *CNTK*. Before installation of *anncolvar* it is necessary to install one of these backends. The package *anncolvar* was tested with *TensorFlow* on a laptop, personal computer and HPC cluster, with *Theano* on HPC cluster and with all three backends in continuous integration environment Travis-CI. Installation of other libraries may be required in order to enable use of GPU acceleration on GPU-equipped computers. Additionally, one needs to install *Python* (*Python* 2.7 and *Python* 3.6 were tested) and package management library *PyPI*.

Once the backend is installed, *anncolvar* can be installed by typing:


pip install numpy cython
pip install anncolvar


(or with *sudo*, depending on user rights and type of installation).

*PyPI* installs all required python libraries. Successful installation can be checked by typing:


anncolvar -h


to print help. *Anncolvar* can be also installed from Anaconda Cloud (https://anaconda.org/spiwokv/anncolvar).

The program *anncolvar* is written in a way so that it requires a preprepared reference structure and a training trajectory. The reference structure is a single structure of the molecular system in PDB format. It is used as a template for RMSD fitting in order to remove translational and rotational motions. Furthermore, input data for artificial neural networks are typically scaled to lie between 0 and 1. The reference structure is used in this process. It must be prepared to fulfill following requirements:

1. It may contain only atoms intended for the analysis. Atoms not intended for the analysis, such as hydrogen atoms, must be deleted. The program *anncolvar* does not ask which atoms are to be analyzed and which are not. Numbering of atoms should not be changed by deletion of unwanted atoms, e.g., if atoms 2, 3, 5, 6, 8, etc. are deleted, the remaining atoms must be numbered 1, 4, 7, etc., not 1, 2, 3, etc.
2. It must be centered in a reasonably large box with coordinates of one corner set to [0,0,0] and the diagonal corner set to [*l*_x_, *l*_y_, *l*_z_] (cubic boxes were used in this work). The size of the box must be sufficient to accommodate the analyzed molecule in all snapshots of the simulation (the program returns an error message if this fails). In the preprocessing step done by *anncolvar* the coordinates are fitted to the reference structure and then divided by *l*_x_, *l*_y_ and *l*_z_ to lie between 0 and 1. The reference structure can be generated, for example, in Gromacs by a command:


gmx editconf -f input.pdb -o reference.pdb -box 6 6 6 -c


for a box with *l*_x_ = *l*_y_ = *l*_z_ = 6 nm. The values of *l*_x_, *l*_y_ and *l*_z_ must be specified by options -boxx, -boxy and -boxz.

The training trajectory must be prepared to fulfill following requirements:

1. It may contain only atoms intended for the analysis, i.e., the same atoms as in the reference structure.
2. The molecule must not be broken due to periodic boundary condition.

Fitting to a template is done by *mdtraj* library in *anncolvar*. For special fitting protocols it is possible to fit the training trajectory before running *anncolvar* and switch off fitting in *anncolvar* by -nofit option.

Finally, the program requires a set of precalculated values of collective variables for each snapshot of the training trajectory (option **-c**). This must be a space-separated file with a column containing values of the CV in the order of snapshots in the training trajectory. The index of the column can be specified by -col (e.g., -col 2 for the second column).

The program makes it possible to modify the design of the neural network, namely the number of hidden layers (1, 2, or 3 is supported), activation functions in each layer (keras activation functions are supported), and the details of optimization (loss function, batch size and optimization algorithm). The results are written to a text output file for easy visualization of the correlation between original and predicted CV values. This output file controlled by **-o** option contains predicted and original values in the first and the second column, respectively. The third column indicates whether the value was used in the training (TR) or test (TE) set. Stratification of data into the training and test sets is controlled by -test (size of test set) and -shuffle (whether snapshots of the trajectory are or are not shuffled before the stratification).

Input file for the PLUMED open-source library for analyzing and biasing molecular dynamics simulations (Tribello et al., 2014) is also provided (-plumed option). This file (default name *plumed.dat*) makes it possible without much changes to calculate the CV for a trajectory (by PLUMED driver) or to monitor the value of the CV during a simulation. Application of the output PLUMED file in metadynamics or other enhanced sampling method supported by PLUMED requires minor changes easy for an experienced PLUMED user. In case the training trajectory and the biased simulation use a different atom numbering, it is necessary to renumber atoms in the PLUMED input file. The reference file is used as a template for fitting of the molecule in order to remove rotational and translational degrees of freedom. It may be necessary to modify the PDB format to fulfill the requirements of PLUMED.

Proper function of *anncolvar* can be checked by recalculation of the CV in the training trajectory using *plumed driver* utility followed by comparison with the text output of *anncolvar*.

A sample training may be executed by:


anncolvar -i traj.xtc -p reference.pdb -c results_isomap -col 2 \
-boxx 1 -boxy 1 -boxz 1 -layers 1 -layer1 64 -epochs 2000 \
-o corr1.txt -plumed plumed1.dat


This carries out 2,000 epochs of training on an artificial neural network with the training trajectory in *traj.xtc* (Gromacs format), reference structure in *reference.pdb* and precalculated CV values in *results_isomap* (in the second column). The artificial neural network was composed of one hidden layer with 64 neurons with sigmoid (default) activation function.

### Simulation Details

All simulations were carried out in Gromacs 5.1.1 (Abraham et al., 2015) with PLUMED 2.4 (Tribello et al., 2014).

Cyclooctane derivative (*trans*,*trans*-1,2,4-trifluorocyclooctane) was simulated as described elsewhere (Spiwok and Králová, 2011). Briefly, it was simulated in General AMBER force field (Wang et al., 2004) in vacuum using stochastic dynamics integrator with 1 fs step and without constraints. Temperature was kept constant at 300 K using Parrinello-Bussi thermostat (Bussi et al., 2007). Electrostatics was modeled without cut-off. The set of 8,375 reference structures was kindly provided by Brown and co-workers (Brown et al., 2008). They were generated by Brown and co-workers using a systematic generation algorithm as described in their work (Brown et al., 2008).

Trp-cage was modeled using Amber99SB-ILDN (Lindorff-Larsen et al., 2010) force field. The protein was placed in a periodic box of size 7 × 7 × 7 nm (metadynamics, MTD) or 3.548 × 3.896 × 3.389 nm (parallel tempering metadynamics, PT-MTD) containing 11,128 (MTD) (Laio and Parrinello, 2002) or 1,366 (PT-METAD) (Bussi et al., 2006) water molecules and one chloride anion. Step of molecular dynamics simulation was set to 2 fs. All bonds were constrained. Electrostatics was modeled by Particle-mesh Ewald method (Darden et al., 1993). Temperature was kept constant using Parrinello-Bussi thermostat (Bussi et al., 2007).

For MTD, the system was minimized by steepest descent algorithm. This was followed by 100 ps simulation in NVT and 100 ps simulation in NVT ensemble. This was followed by 100 ns well tempered metadynamics (Barducci et al., 2008) at 300 K.

For PT-MTD, the system was minimized by steepest descent algorithm. This was followed by 100 ps simulation in NVT and 100 ps simulation in NVT ensemble. The system was preequilibrated by 500 ps NVT simulations at 32 temperatures: 278.0, 287.0, 295.0, 303.0, 312.0, 321.0, 329.0, 338.0, 346.0, 355.0, 365.0, 375.0, 385.0, 396.0, 406.0, 416.0, 427.0, 437.0, 448.0, 459.0, 470.0, 482.0, 493.0, 505.0, 517.0, 528.0, 539.0, 551.0, 562.0, 573.0, 584.0, and 595.0 K. After that PT-METAD was performed at same temperatures. Replica exchange attempts were made every picosecond.

Trajectory of 208 μs simulation of Trp-cage folding/unfolding was kindly provided by D. E. Shaw Research (Darden et al., 1993). It was converted to Gromacs format and prepared by Gromacs tools for analysis in *anncolvar*.

## Results and Discussions

### Cyclooctane Derivative Conformational Transitions

**Figure 2:**
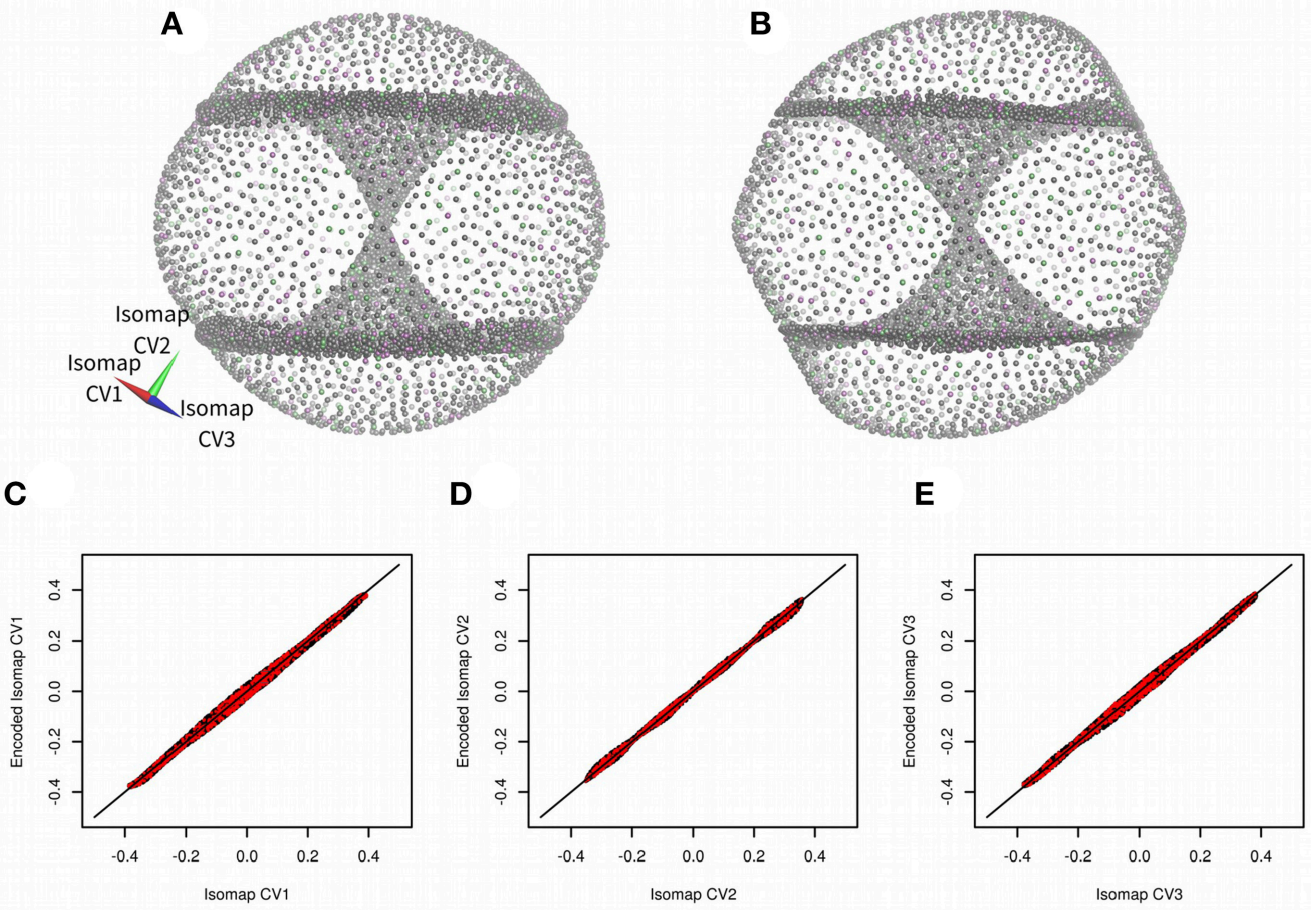
Three-dimensional Isomap embeddings of *trans*,*trans*-1,2,4-trifluorocyclooctane **(A)** and its approximation using *anncolvar* **(B)**. Isomap embeddings in **(A)** and **(B)** were rotated by angle [0.00 rad, 2.40 rad, −0.55 rad] for better clarity. Training set points are in gray, test set points are in different colors depending on whether they were used to train Isomap embedding 1, 2, or 3. Comparison of Isomap embeddings 1 **(C)**, 2 **(D)**, and 3 **(E)** original (horizontal) vs. approximated by *anncolvar* (vertical). Training set points are in black, test set points are in red. Distribution of differences between original and predicted CVs can be found in Figure S1.

Cyclooctane non-symmetric derivative (*trans*,*trans*-1,2,4-trifluorocyclooctane) was introduced as a model molecular system by Brown and co-workers (Brown et al., 2008; Martin et al., 2010). They generated more than one million of conformations of this molecule by a systematic geometry-based algorithm. Then they filtered this set to obtain a set of 8,375 non-redundant structures. These structures were analyzed by a non-linear dimensionality method Isomap (Tenenbaum et al., 2000). Brown and co-workers demonstrated that it is possible to describe conformation of the model molecule using just three low-dimensional Isomap embeddings (see Figure 2A for the reproduction of the results of Brown and co-workers) (Brown et al., 2008).

It is very challenging to use low-dimensional embeddings as CVs in biased simulations. For this, it is necessary to calculate a low-dimensional embedding for a new out-of-sample structure. Furthermore, in order to apply biasing forces on a molecular structure it is necessary to calculate derivatives of the low-dimensional embedding with respect to the Cartesian coordinates. Unfortunately, using Isomap and most other non-linear methods it is not possible to directly calculate neither low dimensional embeddings for a new out-of-sample structure, nor their derivatives. For this purpose we have tested the Property Map Collective Variables (Spiwok and Králová, 2011), an extension of Path Collective Variables (Branduardi et al., 2007). An interesting alternative is application of autoencoders recently used by Chen and Ferguson (2018).

Here we test an artificial neural network performed by *anncolvar* to approximate Isomap embeddings. The set of 8,375 structures provided by Brown et al. (2008) was analyzed by Isomap to obtain three low-dimensional embeddings (Figure 2A). Next we use them to train a neural network to approximate these embeddings. Briefly, we used the command:


anncolvar -i traj_fit.xtc -p reference.pdb \
-c results_isomap -col 2 \
-boxx 1 -boxy 1 -boxz 1 \
-layers 3 -layer1 8 -layer2 8 -layer3 8 \
-actfun1 sigmoid -actfun2 sigmoid -actfun3 sigmoid \
-optim adam -loss mean_squared_error \
-epochs 1000 -batch 256 \
-o low1.txt -plumed plumed1.dat


The set of 8,375 structures was stored in Gromacs format in *traj_fit.xtc*. A reference structure was stored in the file *reference.pdb*. It was centered in the cubic box of size 1 nm with the corners at [0,0,0], [0,0,1], … [1,1,1] (in nm). Isomap low-dimensional embeddings were stored in the file *results_isomap* (space-separated, with structure ID and Isomap embedding 1, 2, and 3 in each column). This carried out 1,000 epochs of training (ADAM optimizer, mean square error loss function) of a network composed of an input layer with 72 neurons (for Cartesian coordinates of 24 atoms) and three hidden layers, each with eight neurons with the sigmoid activation function. By default, 10% of randomly selected structures are used as the test set and remaining as the training set.

This was repeated for the second and third Isomap coordinates (with -col 3 and 4, respectively). The resulting PLUMED input files were combined manually to one PLUMED input file. It was also necessary to renumber atoms due to a different numbering in the original data set and used force field.

There were visible differences between original Isomap embeddings and values approximated by *anncolvar* (Figure 2), nevertheless, these differences do not affect the functionality of embeddings. Pearson correlations of original and anncolvar-predicted Isomap low-dimensional embeddings were higher than 0.997. There was no significant difference between correlations in the training and test sets.

**Figure 3:**
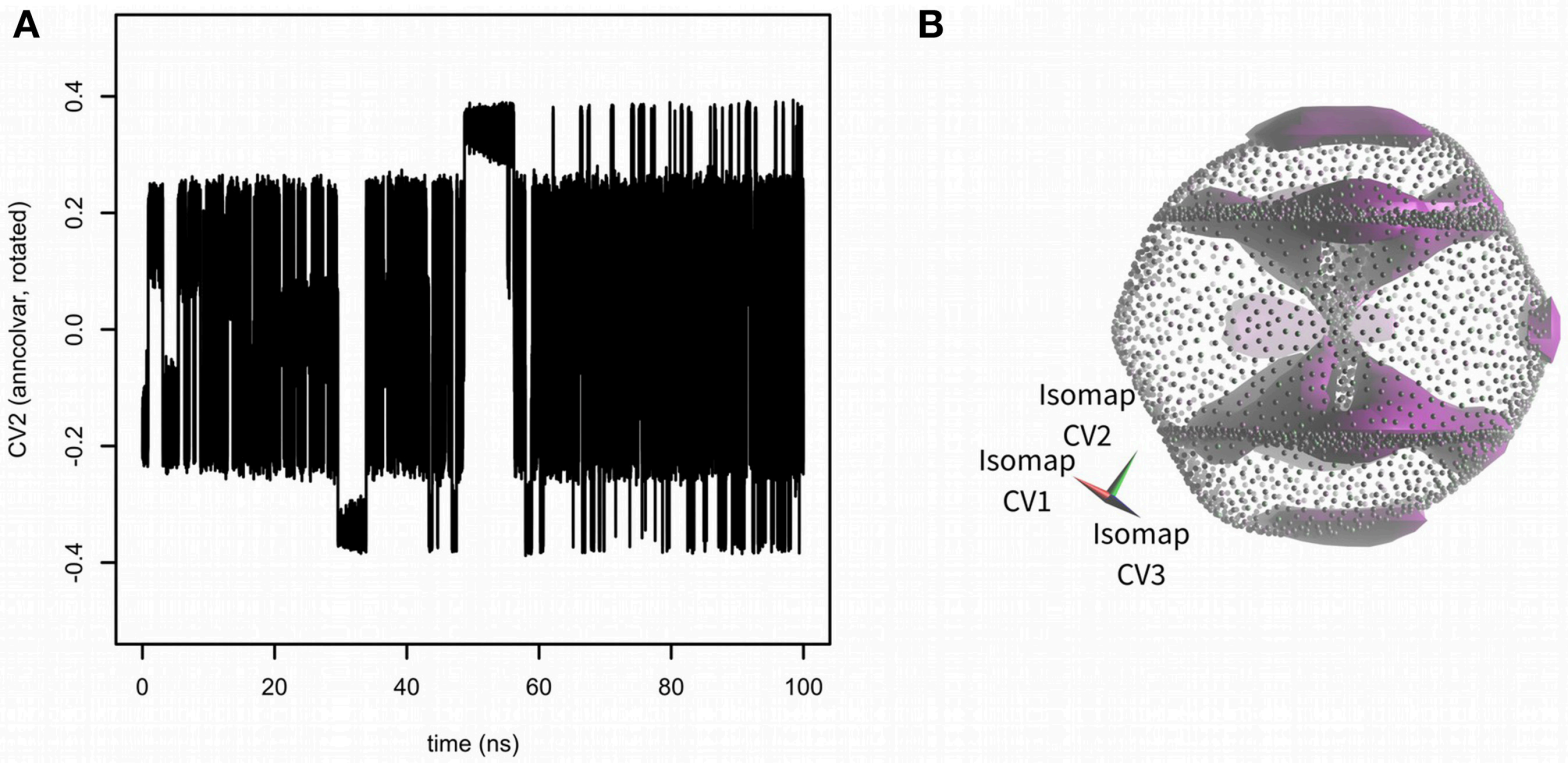
Sampling of CVs in 100 ns metadynamics with Isomap low-dimensional embeddings calculated by *anncolvar* **(A)**. Free energy surface depicted as an isosurface (in violet) at + 30 kJ·mol^−1^ (relative to the global free energy minimum) **(B)**. Isomap embeddings were rotated by angle [0.00 rad, 2.40 rad, −0.55 rad] for better clarity.

Next, the PLUMED input file was edited to enable metadynamics (Laio and Parrinello, 2002) with all three Isomap embeddings used as CVs. Hills were added every 1 ps with constant height of 0.2 kJ·mol^−1^ and width 0.02 (for all three Isomap CVs). The results of 100 ns metadynamics are depicted in Figure 3. The simulation started from one of boat-chair conformation located in the central “hourglass.” After ~20 ns all eight boat-chair conformations were flooded and the system started to explore one of boat conformations at the “equator.” After ~30 ns it started to explore the crown conformation at the “south pole.” At time ~50 ns also the inverted crown at the “north pole” was sampled. The convergence was assessed as the evolution of free energy difference between crown and boat-chair (see Figure S2). The free energy surface was visualized by Mayavi (Ramachandran and Varoquaux, 2011) and PoVRay (Persistence of Vision, 2018)^2^. The resulting free energy surface (Figure 3B) is in good agreement with the results of our previous studies (Spiwok and Králová, 2011; Pazúriková et al., 2017).

### Trp-Cage Folding

Intuitively solvent-accessible surface area (SASA) of a protein is likely to be an interesting CV for protein folding simulation, because SASA of a protein in the folded state is likely to be smaller than for the unfolded state, which is one of requirements for a CV to be successful. For this purpose we used a 208-μs trajectory of Trp-cage miniprotein kindly provided by D. E. Shaw Research (Lindorff-Larsen et al., 2011). We admit that this is not solution to the “chicken-and-egg problem” [as discussed by (Chen and Ferguson, 2018)], because we cannot train the neural network without a long simulation trajectory with folding and unfolding events. Reinforcement learning (Nandy and Biswas, 2018) may be solution to this problem, but it is out of scope of this manuscript.

**Figure 4:**
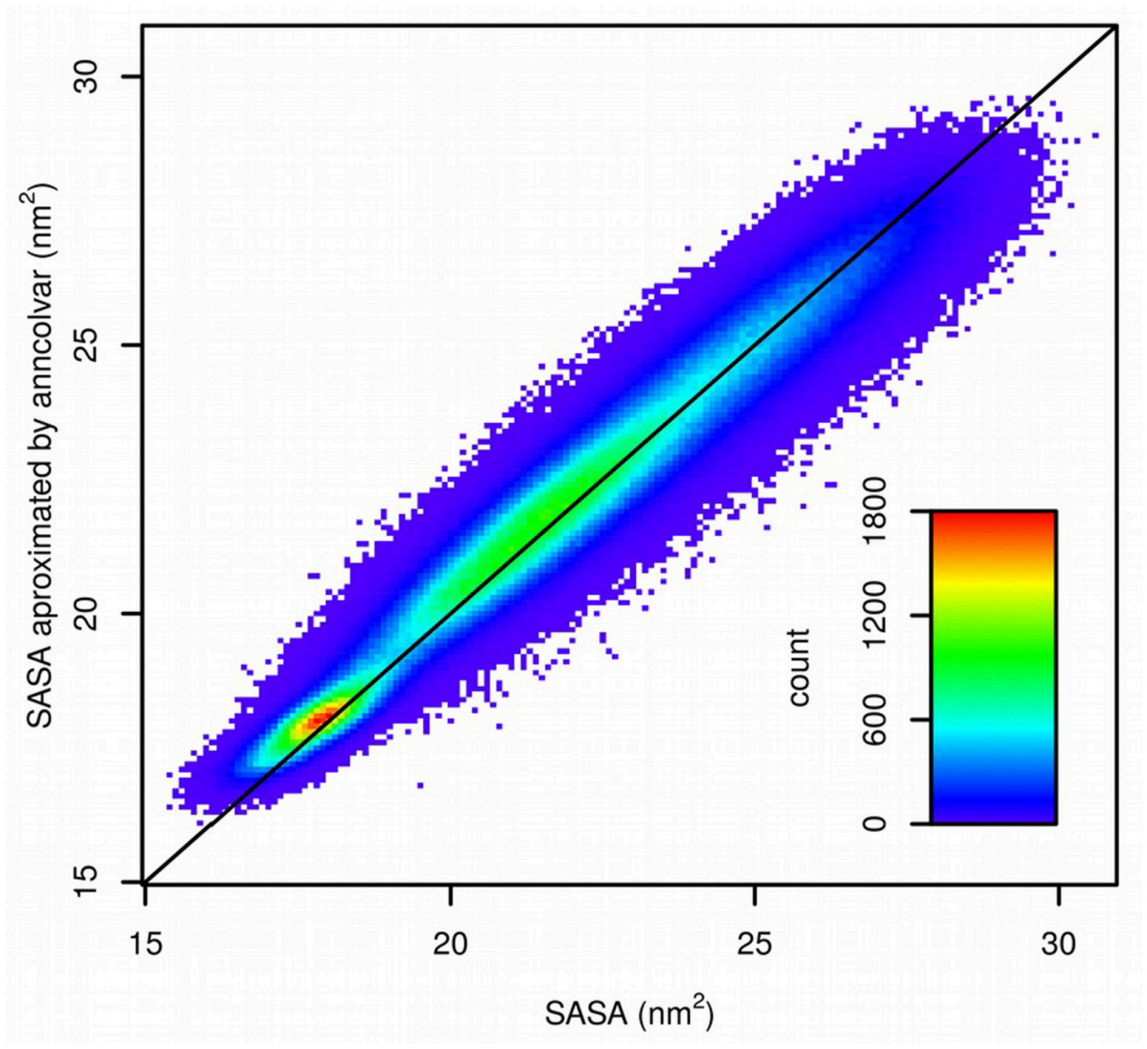
Comparison of SASA calculated by *gmx sasa* (horizontal) and predicted by *anncolvar* (vertical). The line shows the diagonal (*y* = *x*).

The trajectory provided by D. E. Shaw Research was converted to Gromacs format and SASA was calculated for 1,044,000 frames using *gmx sasa* tool from the Gromacs package (Abraham et al., 2015). Next, a neural network was trained in *anncolvar* to approximate SASA. It contained 432 neurons in the input layer (for coordinates of 144 atoms placed in a cubic box of size 6 nm) and one hidden layer with 32 sigmoid neurons. The set of 10% of randomly selected structures was used as the test set and remaining as the training set. This provided a good 0.976 correlation (Pearson) between SASA calculated by *gmx sasa* and predicted by *anncolvar* (Figure 4).

We also examined the effect of training set size on *anncolvar* performance. The observed effect was small. The Pearson correlation coefficient for reference and predicted values ranged from 0.9750 (50% of trajectory frames used) to 0.9756 (90% of trajectory frames used), both using 1,000 epochs. We also tested training using a sub-optimal training set. Unfolded structures (RMSD form NMR structure >0.25 nm on all atoms, 879,759 structures) were selected from the trajectory and used as a training set. The resulting neural network predicts SASA with relatively good accuracy (Pearson correlation coefficient 0.96 for all structures and 0.77 for folded structures, see Figure S7). We plan to test *anncolvar* trained on sub-optimal training sets in future.

In order to evaluate performance of *anncolvar* we decided to estimate costs of SASA calculation by conventional program (*gmx sasa* from Gromacs package) and to compare it with *anncolvar*. The program *gmx sasa* calculates SASA of Trp-cage in approximately one millisecond. This corresponds to reasonably good performance of ~0.6 s/ps or 10 min/ns. However, for biasing it is necessary to calculate not only SASA, but also its derivatives *d*SASA/*dx*. Methods for calculation of analytical surface derivatives have been reported in literature (Sridharan et al., 1995), but their implementation into available simulation packages would require intensive coding. In order to use numerical derivatives it would be necessary to evaluate delta SASA for incremental changes Δ*r* of all coordinates of all atoms. This would downgrade performance to ~days/ns. There are approaches that can be applied to address this problem, such as evaluation of CVs in multiple time steps (Ferrarotti et al., 2015), parallelization or GPU offloading. However, all these approaches either require intensive changes in a code or they may have other disadvantages.

The PLUMED input file was used to drive metadynamics (Laio and Parrinello, 2002) and parallel tempering metadynamics (PT-METAD) (Bussi et al., 2006) with SASA as a collective variable. Similarly to cyclooctane derivative it was necessary to manually edit *plumed.dat* file because of different atom numbering in the D. E. Shaw Research data set and the force field we used. Since formation of secondary structure is very important and potentially the slow step of Trp-cage folding, another CV was used to enhance formation of secondary structure. We selected an alpha helical content of a protein structure (ALPHARMSD) (Pietrucci and Laio, 2009) collective variable with parameters set to default in PLUMED. Well-tempered metadynamics was performed with hills of height 1 kJ·mol^−1^ added every 1 ps with hill widths 1 nm^2^ for SASA and 1 for ALPHARMSD, respectively. Bias factor of well-tempered metadynamics was set to 15 (Barducci et al., 2008). Unfortunately, 100 ns metadynamics starting from the folded structure lead to quick unfolding but not to folding (see Supporting Information, Figure S3).

Therefore, in order to enhance sampling in degrees of freedom that cannot be addressed by the applied CVs we replaced metadynamics by PT-METAD (Bussi et al., 2006). The system was simulated at 32 temperatures ranging from 278.0 to 595.0 K. Metadynamics parameters were not changed. The plot in Figure S4 demonstrated significant overlap of potential energy histograms, which is a prerequisite for a reasonable replica exchange rate. During a PT-METAD (50 ns in each replica) we observed eight folding events (recognized by visual inspection of “demuxed” trajectories, see Supporting Information, Figure S5). This is in contrast to a parallel tempering molecular dynamics simulation with otherwise same parameters (without metadynamics), where no folding events were observed.

The size of box in PT-METAD was small to increase replica exchange probability and thus to reduce required number of replicas. We admit that this increases risk of self-interaction artifacts in folding simulations. We visually examined folding simulation trajectories and discovered examples of self-interactions (see Figure S6). These interactions were relatively short-living. Moreover, we believe that self-interactions complicate, not facilitate, folding. Therefore, neural network approximated SASA can be seen as a successful CV.

**Figure 5:**
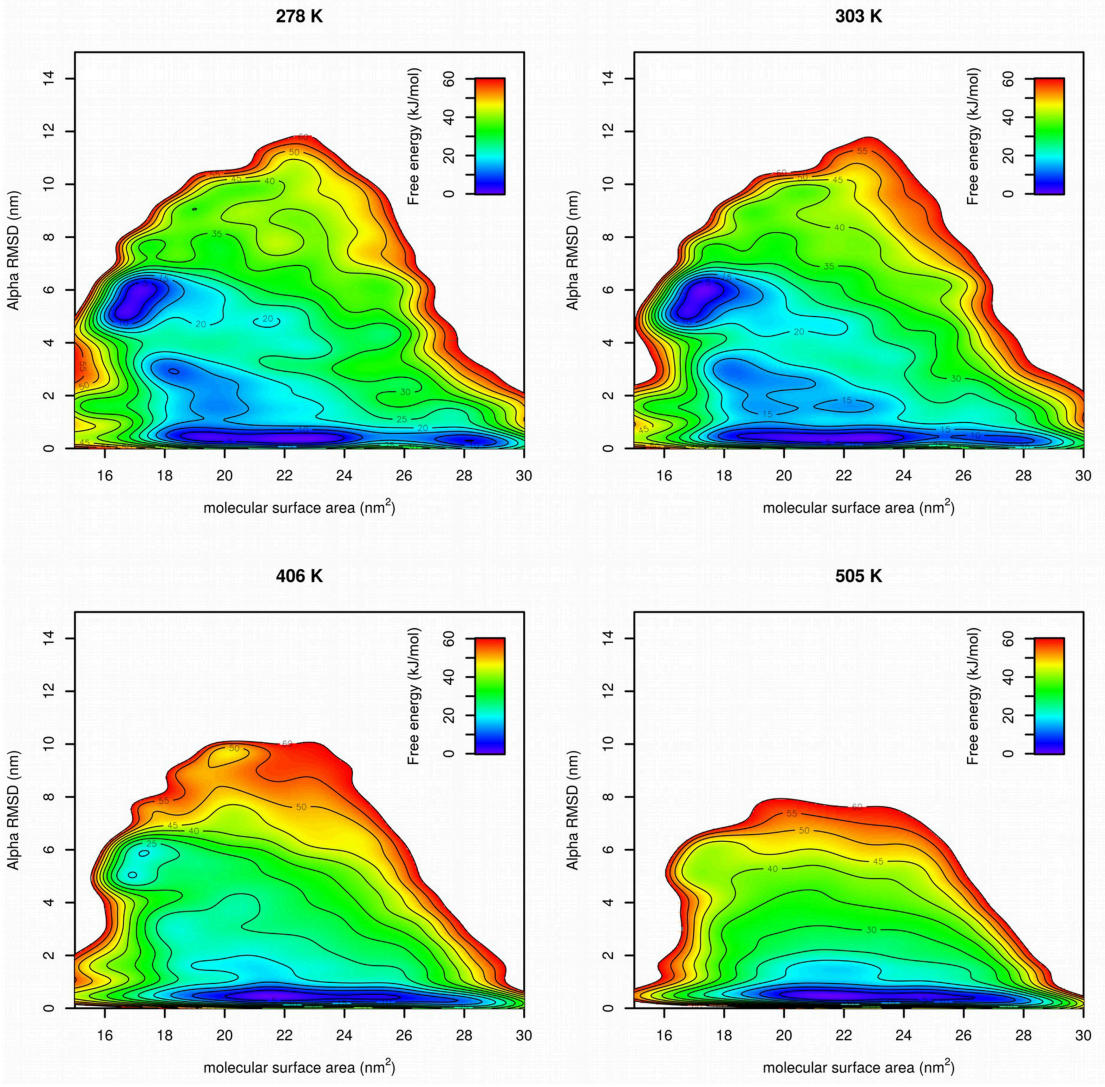
Free energy surfaces of Trp-cage calculated by PT-METAD with SASA and Alpha RMSD collective variables at four selected temperatures.

Free energy surfaces were calculated from Gaussian hills accumulated at each temperature in PT-METAD (Figure 5). Free energy surfaces are in a good agreement with the results from literature (Lindorff-Larsen et al., 2011). At low temperatures there were two free energy minima with approximately same value of free energy. One at CVs [~17 nm^2^, ~6] corresponds to the folded structure. The second one at CVs [~21 nm^2^, ~0.5] corresponds to the unfolded structure. The fact that both minima have approximately same free energy value is in agreement with the fact that in an unbiased simulation (Lindorff-Larsen et al., 2011) the system spends approximately same time in unfolded and folded state. At slightly elevated temperatures the minimum corresponding to the folded state becomes more shallow and at high temperature it becomes almost indistinguishable. Other states, such as those with higher helical content or low-SASA states with low helical content, were predicted as energetically unfavorable.

One of the motivations for development of *anncolvar* was the potential speed gain compared to Path Collective Variables and Property Map. These two approaches require multiple RMSD-fitting processes in each step. This problem has been addressed by Close Structure algorithm (Pazúriková et al., 2017), which reduces the number of RMSD-fitting processes, but still requires multiple RMSD-fitting processes in some steps of the simulation. The approach presented here requires only one RMSD-fitting in each step. RMSD-fitting free approaches (such as those using interatomic distances) are not supported by *anncolvar*, but can be used in future if it turns out to be a viable strategy.

For the cyclooctane derivative, metadynamics was significantly slower than unbiased simulation (~40 ns·day^−1^ vs. ~7 μs·day^−1^ on single CPU). However, this can be explained by the fact that not only CV calculation, but also calculation of the bias potential takes large proportion of CPU load in the system much smaller (24 atoms) than biomolecular systems with explicit solvents. On the other hand, the situation was much more favorable in biomolecular systems with an explicit solvent. Metadynamics (Trp-cage with 11,128 water and one chloride) was approximately twice slower than corresponding unbiased simulation (both on 8 CPU cores). Examination of one part (5 ns) of metadynamics simulation revealed that metadynamics force calculation accounts for 78% of total force calculations and 59% of total calculations. Similarly PT-METAD (Trp-cage with 1,366 waters and one chloride) was also approximately twice slower than corresponding unbiased parallel tempering simulation (both on 32 CPU cores).

Neural networks architectures used in this study were relatively small to avoid slowing down of simulations. They are not deep enough to be called deep learning. There are several options to improve the program in order to enable deeper neural network models. For example, we plan to enable loading of weights and biases into PLUMED as text files. This would also simplify file handling. There is also space for parallelization and GPU offloading. We plan to work on this in near future.

In this work we used two different data sets to train the neural network. The first was generated by a systematic conformer generation. The second was generated by a long molecular dynamics simulation. Both approaches require that the structure corresponding to the free energy minimum is present in the training data set. This leads to the “chicken-and-egg problem” discussed by Chen and Ferguson (Chen and Ferguson, 2018). We have to know the structure of folded protein (or at least it must be present in the training data set without knowing that it is the folded one) in order to simulate folding of the protein. Therefore, the approach outlined in this work is suitable to study protein folding mechanisms with known folded structure, but it is not suitable for *de novo* structure prediction. Generative machine learning models, which involve models that can make accurate prediction outside the training set as they learn a broad distribution of the training set, may be useful to address this problem. Reinforcement learning can be another answer to this problem.

## Author Contributions

All authors contributed to the code of anncolvar. DT, IH, and VM: tested the code; DT, IH, and VS: carried out simulations, all authors contributed to the manuscript.

### Conflict of Interest Statement

The authors declare that the research was conducted in the absence of any commercial or financial relationships that could be construed as a potential conflict of interest.

## Footnotes

^1^Cholet, F., and co-workers, https://keras.io, 2019.

^2^Persistence of Vision Pty. Ltd., http://www.povray.org, 2018.
