# Supplementary material for: Anncolvar: Approximation of Complex Collective Variables by Artificial Neural Networks for Analysis and Biasing of Molecular Simulations
Source: Front Mol Biosci. 2019 Apr 18;6:25. doi: 10.3389/fmolb.2019.00025 (PMC6482212; doi:10.3389/fmolb.2019.00025)
Supplement: Supplementary file 1 [file Data_Sheet_1.PDF]

Supporting information

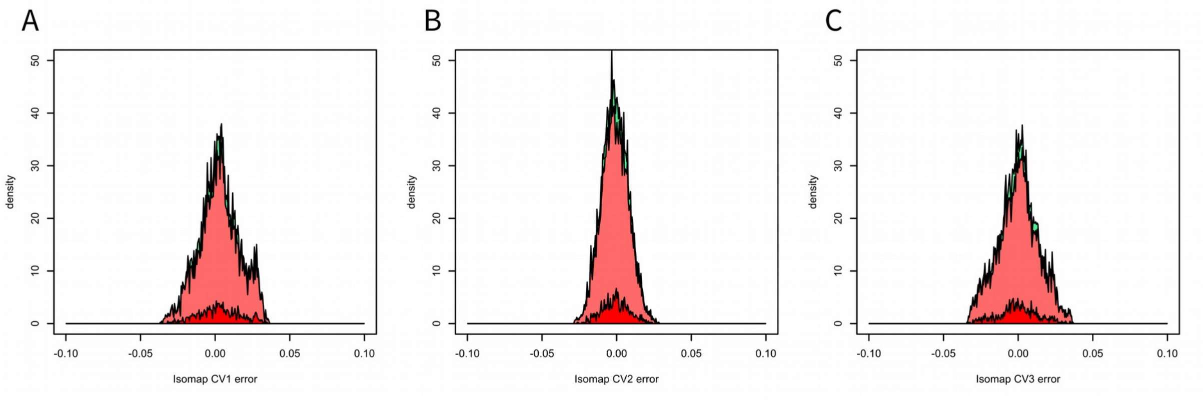

Figure S1: Density plots of errors of Isomap CVs (predicted–reference, **A** - CV1, **B** - CV2 and **C** - CV3). All values are depicted in green, training set is in salmon and test set in red.

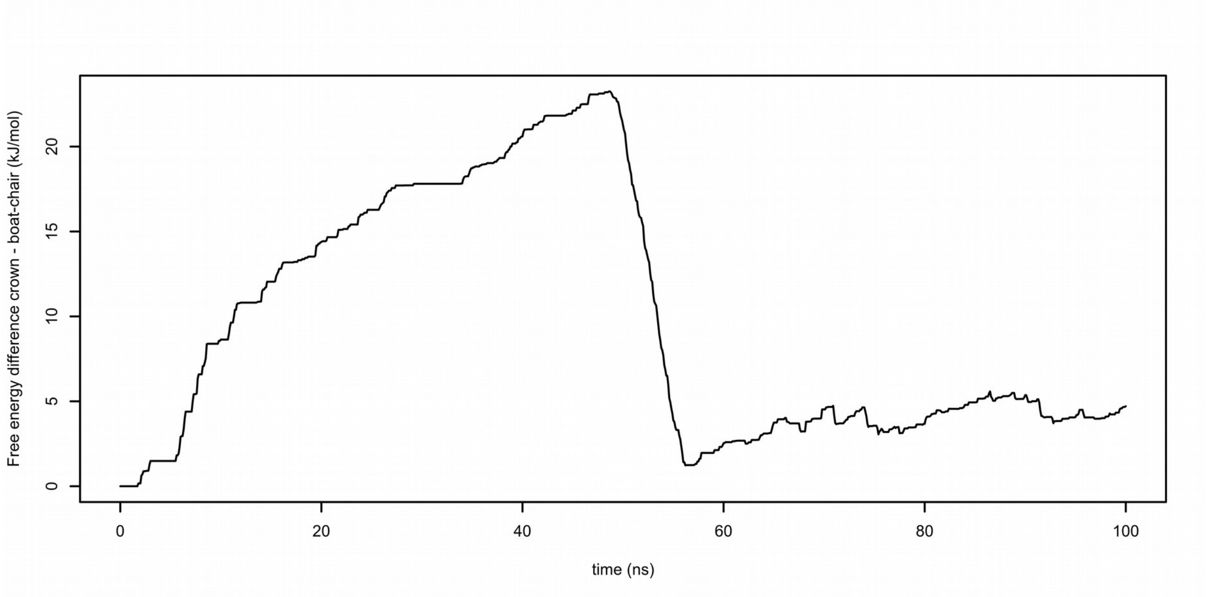

Figure S2: Evolution of free energy difference between crown and boat-chair conformations of the cyclooctane derivative.

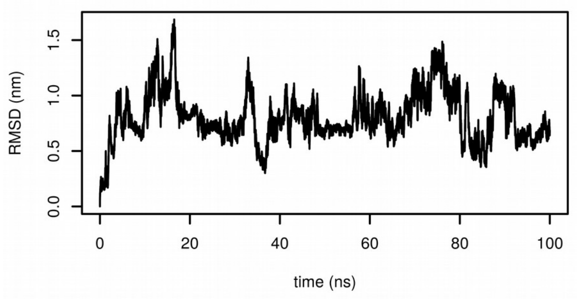

Figure S3: Evolution of root-mean-square deviation from the native structure during metadynamics. It shows unfolding in less than 5 ns but does not show folding.

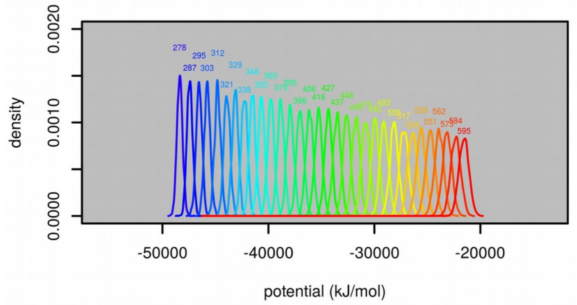

Figure S4: Potential energy density in equilibration simulations preceding parallel tempering. It shows that there is a substantial overlap between neighboring replicas, which indicates reasonable exchange rate.

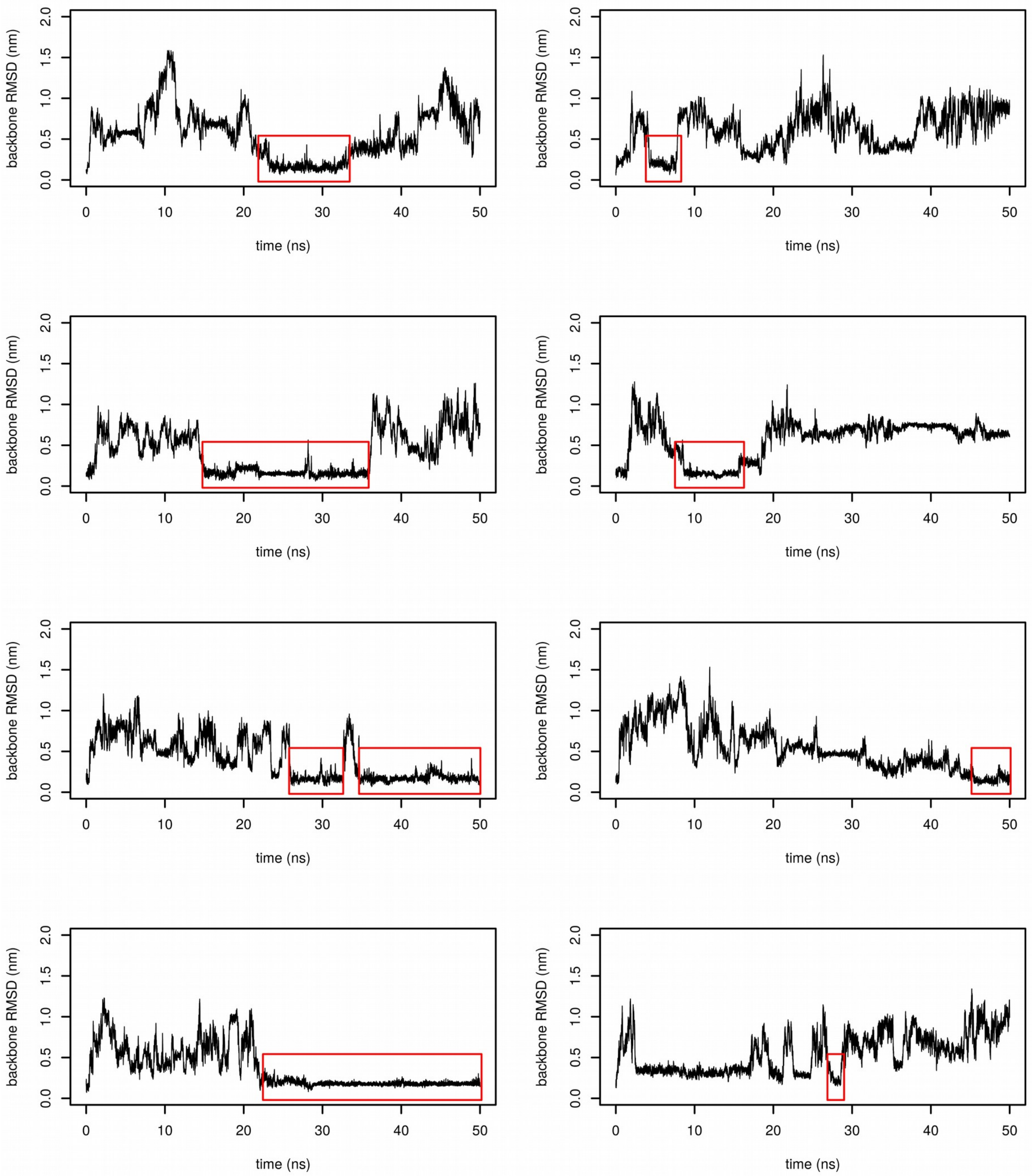

Figure S5: Root-mean-square deviations from the native structure in demuxed replicas showing folding in parallel tempering metadynamics. Folded snapshots are highlighted by red boxes.

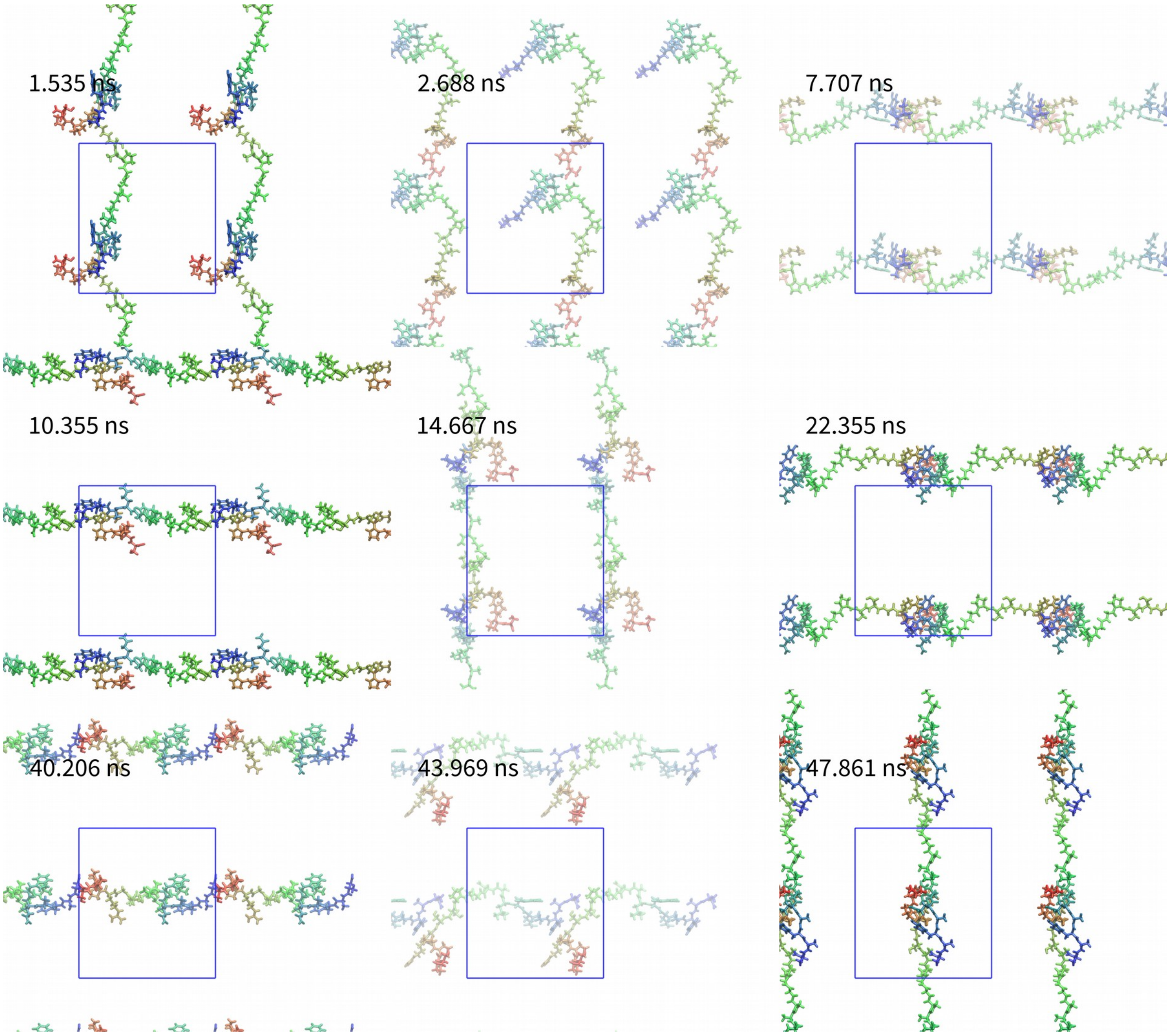

Figure S6: Examples of self-interactions observed in sampling at lowest temperature in PT-METAD. The fraction of self-interacting structures was small and these structures were short-living.

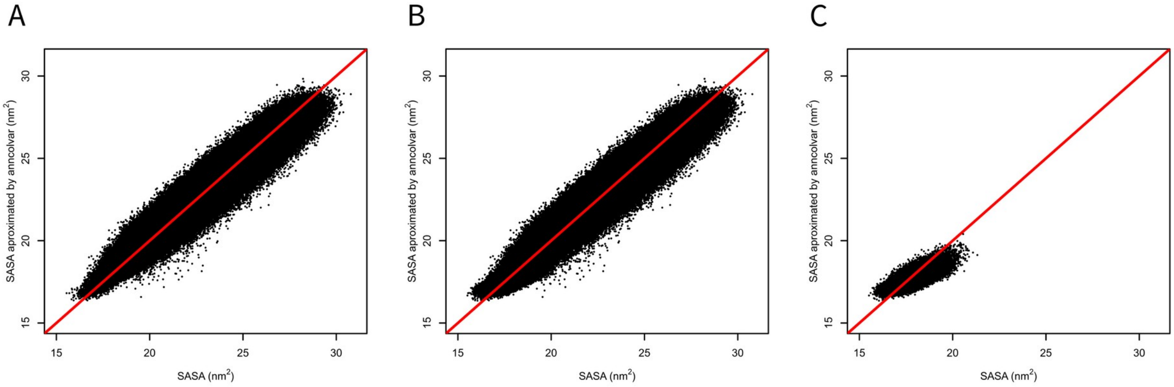

Figure S7: Training of *anncolvar* on a sub-optimal training set. Unfolded structures (RMSD on all atoms > 0.25 nm, 879,759 structures) were selected from the trajectory and used as a training set. The results of training are depicted as a correlation between predicted and reference SASA for training set (**A**, Pearson correlation coefficient 0.96), all snapshots (**B**, Pearson correlation coefficient 0.96) and folded structures (**C**, Pearson correlation coefficient 0.77).
